# Exercise and Sport Participation in Pediatric Hypertrophic Cardiomyopathy: A Changing Paradigm

**DOI:** 10.1007/s11936-026-01150-5

**Published:** 2026-08-03

**Authors:** William W. Russell, Jonathan B. Edelson

**Affiliations:** Department of Pediatric Cardiology, Children’s Hospital of Philadelphia, 3401 Civic Center Blvd, Philadelphia, PA 19104 USA

**Keywords:** Hypertrophic cardiomyopathy, Exercise, Sports, Shared decision-making, New guidelines

## Abstract

**Purpose of Review:**

This review aims to detail recent research and guidelines that have changed the approach to exercise and sport participation in the pediatric hypertrophic cardiomyopathy (HCM) population.

**Recent Findings:**

Recent studies, including the landmark LIVE-HCM cohort, demonstrated that vigorous exercise was not associated with death, implantable cardiac defibrillator (ICD) shock, resuscitated cardiac arrest, or arrhythmic syncope in patients with HCM. Furthermore, there is increasing evidence that restriction from physical activity (PA) has negative consequences for the cardiovascular health and emotional well-being of children with HCM. This has influenced guidelines that promote individualized risk assessment and shared decision-making (SDM), rather than uniform restriction, in children with HCM who wish to participate in both recreational and competitive athletics.

**Summary:**

The relationship between PA and cardiac events in children and adults with HCM is more nuanced than once believed, and the negative consequences of uniform PA restrictions have become increasingly clear. Guided by recent prospective cohort studies, the approach to adolescents and young adults with HCM is changing, now emphasizing the role of individualized risk assessment and SDM.

## Opinion Statement

Prior expert consensus, including the 2014 European Society of Cardiology (ESC) Guidelines on Hypertrophic Cardiomyopathy and the 2015 American Heart Association/American College of Cardiology (AHA/ACC) Task Force 3 recommendations, generally restricted children with hypertrophic cardiomyopathy (HCM) from participation in competitive sports and many forms of recreational physical activity. These recommendations were derived largely from adult observational studies, autopsy registries, and expert opinion rather than pediatric-specific data. However, data suggests that the risk of exertional related cardiac events is likely not as high as once believed, and that restriction is associated with negative cardiovascular and mental health consequences. Although much of the evidence supporting this evolution in orientation originates from adult and mixed-age cohorts, several studies including children (LIVE-HCM) and pediatric-focused expert statements (2026 AHA scientific statement) have reinforced the importance of individualized risk assessment and shared decision-making (SDM) rather than universal restriction. Accordingly, balancing the risks and benefits of physical activity should incorporate both patient-specific risk factors and the physical demands of the desired activity while recognizing the limitations of the currently available pediatric evidence.

## Introduction

Hypertrophic cardiomyopathy (HCM) is the most common inherited cardiomyopathy in children, accounting for 35–50% of cases and is characterized by the presence of ventricular hypertrophy that cannot be attributed to abnormal loading conditions (such as systemic hypertension or congenital aortic stenosis) or secondary causes including infiltrative, metabolic, syndromic, endocrine, or neuromuscular disorders [1]. In children, the diagnosis is most often made with cardiac imaging, with echo and/or cardiac MRI demonstrating abnormal ventricular wall-thickness, frequently including asymmetric thickening of the ventricular septum.

Traditional guidance for exercise in patients with HCM featured restriction [2, 3], due to concerns that athletic participation could precipitate sudden cardiac death (SCD) through either catecholamine surge triggering ventricular arrhythmia or dynamic left ventricular outflow tract (LVOT) obstruction leading to inadequate coronary perfusion. There was also concern that regular physical activity (PA) could worsen disease progression by accelerating pathologic hypertrophy. However, many of these expert recommendations were based on autopsy studies, which likely overestimated the risk of exercise in HCM patients [4]. More recent data has demonstrated that the relationship between HCM and exercise is likely more complicated than previously believed. In a pediatric and young adult cohort, HCM-related mortality has been estimated at 0.5% per year [5]. A pediatric-specific cohort demonstrates cumulative risk of SCD events were: 2.8% at 1 year, 9.1% at 5 years, and 15.0% at 10 years [6]. Furthermore, contemporary studies have not demonstrated an independent association between vigorous exercise and major arrhythmic events [7–9] and preliminary data suggests that exercise may in fact lead to positive cardiac remodeling and improved diastolic performance. There is also accumulating evidence that exercise restriction has a negative effect on emotional and cardiovascular health in children with HCM [10]. This has led to a shift in expert recommendations for patients with HCM [11–13], that emphasizes individualized risk assessment and shared decision making (SDM). This progression is particularly relevant in children given the importance of physical activity during childhood. This review will review the recent data on exercise in HCM and focus on how physical activity related recommendations in HCM are evolving, with an emphasis on shared decision-making (SDM).

## Key Research Findings

Historically, being diagnosed with HCM typically resulted in significant restriction of exercise, with prior guidelines supported by a series of studies that demonstrated an increased risk of cardiac events during PA in patients with HCM [14]. Among these studies was Barry Maron’s paper on sudden death in young athletes (30 years or younger), an autopsy study published in 1980 and showed that HCM was the leading cause of SCD accounting for 14 out of 29 cases (55% 18 years or younger) [4], and his follow up work from the Registry of Sudden Death in Athletes, which found that HCM was again the leading cause of death, accounting for 36% (302 cases) of the 842 cases with confirmed cardiovascular diagnoses (adult/pediatric mixed cohort, mean age 19) [15]. These studies, although limited by ascertainment bias, alongside several high-profile SCD in athletes, led to guidelines that restricted most forms of exercise in young people with HCM. As recently as 2014 and 2015, groups including the European Society of Cardiology, American Heart Association, and American College of Cardiology, all recommended avoidance of most competitive sports for patients with HCM. [2, 3]Often these recommendations were extended by cardiologists to include all forms of exercise, and many patients with HCM were restricted from both recreational and competitive forms of PA [2].

More recently, investigators have acknowledged several limitations of the prior retrospective studies, including the inclusion bias that comes from using an autopsy-based cohort. Furthermore, the mechanism by which many children are diagnosed has changed; no longer are most patients diagnosed with HCM after a cardiac event. Instead, it is our observation that with more widespread implementation of genetic testing and cascade screening, the population of children diagnosed with HCM has shifted to include a larger proportion with a less malignant phenotype, including many who are genotype-positive, phenotype-negative. As such, to better understand the risk of competitive sports in adolescents with HCM, there is a need to risk-stratify patients by phenotypic severity. A study from Pelliccia and colleagues retrospectively evaluated 88 athletes, 61 of whom had significantly reduced their level of athletic fitness (“de-trained”) and found no difference in freedom from sudden cardiac arrest/death or symptoms in the cohort restricted from exercise (adult cohort, mean age 31) [8]. Martinez and colleagues have followed a cohort of NCAA and professional athletes, 72% of the HCM patients were initially disqualified from competitive sport participation and opted to return to play (with SDM and after a comprehensive clinical evaluation). At the time of publishing, there was only 1 exercise-related and 2 non-exercise breakthrough cardiac events (adult cohort, mean age 20) [9]. While these studies are adult studies, the median age is relatively young and suggests that structured evaluation and SDM can support safe return to sport in certain HCM patients.

It was in the setting of an evolving mindset towards HCM and exercise that the first large, prospective cohort study was designed to determine whether vigorous exercise, including competitive sports, increases the risk of life-threatening ventricular arrhythmias and/or mortality in patients with HCM. *Vigorous exercise in patients with hypertrophic cardiomyopathy* (LIVE-HCM) [7] enrolled 1660 participants, with either genotype-positive phenotype-negative HCM or phenotype-positive HCM (adult/pediatric mixed cohort, mean age 39, 13% less than 18 years). Participants were classified into three self-reported levels of PA: sedentary, moderate, or vigorous-intensity exercisers. Among 259 vigorous competitive athletes included in the study, 93 (35.9%) were < 18 years and 31 (12.0%) were 18–25. The primary composite outcome was death, resuscitated sudden cardiac arrest, arrhythmic syncope, and appropriate shock from implantable cardiac defibrillator (ICD). A total of 4.6% of patients reached the composite endpoint but, importantly, in the vigorous-competitive athlete sub-analysis, there was not associated with higher risk (hazard ratio: 0.71; upper 95% one-sided confidence level: 1.32). There were also no events for the genotype-positive phenotype-negative population, providing further evidence that these patients should be routinely monitored, but not routinely restricted from exercise. Unfortunately, a dedicated pediatric analysis of the primary endpoint was not included. However, an abstract published in JACC 2026 by Marino and colleagues, specifically analyzed pediatric patients in the LIVE-HCM study and found that the vigorous exercisers (and their parents) reported high quality of life, as compared to non-vigorous exercisers [16]. This research has directly led to the changes in the current guideline recommendations for exercise in pediatric HCM, discussed in more detail below.

While the historical understanding of the relationship between vigorous exercise and the risk of cardiac events has evolved, there has also been increased attention to the negative consequences of PA restriction on cardiovascular health and mental well-being in both children and adults with HCM. Specifically in the pediatric HCM population, activity restriction and obesity remain a significant problem. In a multicenter cross-sectional survey study of Canadian patients aged 10–19 with sarcomeric HCM, investigators found that 75% of subjects were restricted from PA, and that over half were overweight or obese [17]. On self-report, 96% of patients participated in less than 20 min of exercise per day and had additional unhealthy patterns regarding diet and sleep. The negative impacts of PA restriction have been further reinforced by several additional studies. Balaji and colleagues found that 30% of pediatric HCM patients had BMIs greater than the 99th percentile, and that obesity was associated with an increased left ventricular posterior wall thickness [18], suggesting a potential link between sedentary activity and disease progression in children with HCM.

Importantly, the negative effects of PA restriction appear to extend beyond the cardiovascular system. A study published in 2024 by Wagner and colleagues assessed quality of life (QOL) of patients with HCM in an observational cohort study (pediatric cohort). They found the entire cohort had lower QOL scores, as compared to the reference values, but those who had more daily steps and distance traveled had associated higher QOL scores, emphasizing the bidirectional relationship between PA and quality of life [19]. A separate study of patients with HCM showed that the majority of subjects feel that exercise-restriction has negatively affected their emotional well-being and that there is high prevalence of anxiety related to exercise that likely further contributes to decreased PA levels (adult cohort) [10]. These findings provide further evidence that exercise restriction negatively impacts mental health across patient populations with cardiac disease, including HCM.

It is also important to recognize that patterns of PA vary as a function of socioeconomic disparities. In 2025, Masood and colleagues conducted a retrospective, cross-sectional analysis HCM patients (pediatric cohort) who underwent cardiopulmonary exercise testing (CPET) [20]. From 155 patients, they found that lower child opportunity index, increased urbanization, and lower neighborhood wealth were independently associated with lower exercise performance. These findings are especially important to consider as we move from an era of restriction to one of promotion, and to ensure that the positive effects of PA are equally available to all children with HCM.

### Guideline and Consensus Updates

Improved characterization of pediatric HCM risk, including contemporary estimates of SCD from the PRIMaCY cohort [6], in addition to data that blanket restrictions come with substantial cost to cardiovascular health and emotional well-being, has led to a more nuanced approach to PA in both adults and children with HCM that emphasizes individualized risk assessment and SDM. The 2024 AHA/ACC/AMSSM/HRS/PACES/SCMR guidelines for management of HCM in competitive sports are not pediatric specific. These guidelines recommend yearly evaluation with an HCM specialist if playing sports [11]. Following a comprehensive evaluation, sport participation should be evaluated through a SDM lens that balances potential benefits and risks, a class 2B recommendation. A pediatric-focused JACC expert review from Dean and colleagues agrees with current guidelines that support an exercise prescription that incorporates SDM and risk stratification. They also highlight the utility of using models such as HCM Risk-Kids and PRIMaCY scores as an additional tool to help determine the risk/benefit ratio of sports participation [21]. HCM Risk-Kids estimates 5-year SCD risk using readily available clinical variables including maximal left ventricular wall thickness, left atrial diameter, unexplained syncope, ventricular tachycardia, and peak left ventricular outflow tract gradient [22]. PRIMaCY similarly estimates pediatric SCD risk using a combination of demographic, imaging, ECG, and clinical characteristics from an international pediatric HCM cohort [6]. Importantly, neither model was developed to predict exercise-related risk specifically; rather, they estimate sudden death risk, providing additional context during discussions regarding sports participation.

The 2024 h expert consensus statement on arrhythmias in the athlete provides a class 1 recommendation for return to play in the genotype-positive, phenotype-negative HCM patient [12]. There is a class 2a recommendation for sports participation in phenotype-positive HCM patients, in conjunction with an HCM expert physician, shared-decision-making, and an emergency action plan (EAP). This class 2a recommendation is in slight discordance with the 2024 AHA/ACC/AMSSM/HRS/PACES/SCMR HCM guidelines, which give phenotype-positive patients a Class 2b recommendation for competitive sports. The HRS guidelines also advise against placing an ICD to facilitate return to play and provides a more specific recommendation to “young athletes” with HCM for close follow up and monitoring for progression of disease. The 2025 ACC/AHA scientific statement on sports participation for athletes with cardiovascular abnormalities (adult-focused) supports fewer global restrictions on athletics and sport participation [13]. They also recommend a yearly evaluation and a focus on SDM. A 2026 scientific statement from the AHA is pediatric-focused and largely congruent with the previously cited adult guidelines, encouraging light to moderate-intensity PA, while high-intensity exercise and sport participation are reasonable to consider in certain patients with HCM, with a goal of promoting forms of PA that are compatible with the patient and family desires, after assessing for patient-specific risks [23]. The relevant guidelines are summarized in Table 1.

**Table 1:** Recent Updates for Sports Participation in HCM

New relevant guidelines / Scientific statements	Noteworthy takeaways	Sports recommendation
AHA/ACC/AMSSM/HRS/PACES/SCMR Guidelines for the Management of HCM (2024) [11]	• CPET recommended to determine functional capacity and provide prognostic information • Use SDM in determining sports participation	• Mild-moderate intensity recreation is beneficial (Class 1) • For competitive sports, comprehensive evaluation + SDM with expert professional is recommended (Class 1) • Participation in competitive sports is reasonable, after evaluation and SDM (Class 2b)
2024 h Expert Consensus Statement on Arrhythmias in the Athlete* [12]	• No indication for sports restriction on genotype-positive, phenotype-negative patients • Recommend against placing ICD to facilitate return to play • Use SDM in determining sports participation	• Genotype-positive phenotype-negative can play competitive sports in conjunction with expert assessment (Class 1) • If obstructive HCM, intentional measures to attenuate LVOT obstruction should be made prior to return to play (Class 1) • Phenotype-positive HCM participation in sports is reasonable with appropriate therapy and EAP (Class 2a)
AHA/ACC Scientific Statement for Clinical Considerations for Competitive Sports Participation for Athletes with Cardiovascular Abnormalities (2025) [13]	• Recommends fewer global restrictions on sport participation • Yearly evaluation from HCM specialist with focus on SDM	N/A
AHA Scientific Statement: Physical Activity in Pediatric Cardiomyopathies (2026)* [23]	• Encourage light-moderate intensity exercise • Higher intensity exercise and sports participation to be considered with SDM	N/A
The Cardiovascular Care of the Pediatric Athlete (JACC Expert Review − 2025)* [21]	• Risk calculators (HCM Risk-Kids and PRIMaCY) can be useful additional tool to help inform SDM conversation	N/A

* Contains pediatric-specific exercise recommendations

*ACC *American College of Cardiology, *AHA *American Heart Association, *AMSSM *American Medical Society for Sports Medicine, *CPET *cardiopulmonary exercise testing, *EAP *emergency action plan, *HCM *Hypertrophic Cardiomyopathy, *HRS *Heart Rhythm Society, *ICD * implantable cardiac defibrillator, *JACC *Journal of the American College of Cardiology, *LVOT *left ventricular outflow tract, *PACES *Pediatric and Congenital Electrophysiology Society, *SCMR *Society for Cardiovascular Magnetic Resonance, *SDM *shared decision-making

Alongside the evolving approach to PA in children with HCM, there has also been an increased emphasis on the use of CPET, both to provide an objective assessment of functional status, and to inform individual risk. The new 2024 ACC/AHA/AMSSM/HRS/PACES/SCMR HCM guidelines specifically state that CPET is recommended for all pediatric HCM patients to provide a functional baseline and assess for ischemic symptoms or ECG changes, which can provide prognostic information [11]. These guidelines are supported by recent studies identifying CPET markers associated with clinical outcomes. Conway and colleagues conducted an international retrospective study of 630 phenotype-positive HCM patients, examining whether abnormal exercise response — defined as complex ventricular ectopy, ST segment changes, or blunted blood pressure response — was associated with adverse outcomes (pediatric cohort) [24]. Those with an abnormal exercise response demonstrated greater septal hypertrophy, higher left atrium diameter z-scores, higher resting LVOT gradients, and higher rates of myectomy. Furthermore, an abnormal CPET was associated with a lower 5-year transplant-free survival, especially those with exercise-induced ischemia. Another retrospective study, performed by at the Children’s Hospital of Philadelphia, included 140 patients [25] and found that ectopy on CPET was an independent risk factor for a composite endpoint that included cardiac death, transplant, or arrhythmia requiring ICD placement (pediatric cohort). Both studies conclude, and the guidelines now agree, that there is a prognostic indication for routine CPET in pediatric HCM patients.

## Clinical Practice Evolution

**Fig. 1:**
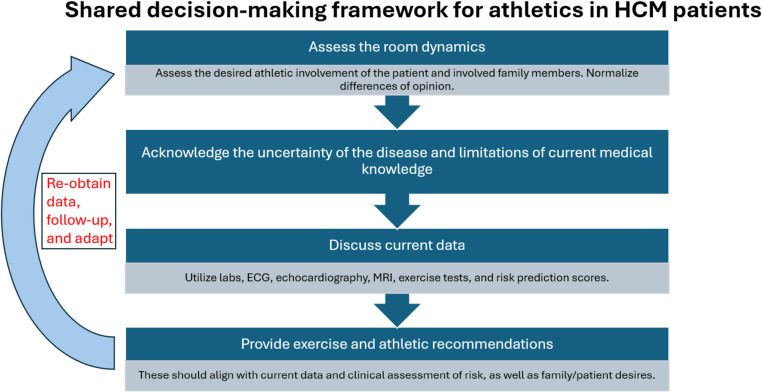
Shared decision-making framework for athletics in HCM patients. (From: Russell WW, et al. JACC Case Rep. 2026;31(12). doi:10.1016/j.jaccas.2026.107154, with permission from Elsevier) [23]

A major challenge in pediatric HCM management relates to balancing the rarity of exercise-associated sudden death against the difficulty in determining the risk-benefit ratio. With new exercise guidelines focusing on SDM, groups have published strategies for the physician approach to exercise discussions and re-evaluation. Semsarian and colleagues created a stepwise decision-making process for physicians [26]. Their proposed evaluation included risk stratification, patient and family education, determination of patient values, engagement of stakeholders (patient, parents, team, other treating physicians), and longitudinal follow-up. We propose a similar framework to promote the physician’s use of SDM in pediatric HCM patients (Fig. 1) [27].

1. Assess the room dynamics. Often the patient will come to the clinic visit with both parents, this allows for three (possibly entirely different) viewpoints. After meeting everyone, it is important to elicit the desired level of athletic involvement and any reservations, from each stakeholder. Differing opinions, as is often the case, should be normalized.
2. Acknowledge uncertainty of the disease and limitations of current medical knowledge. HCM is a severe disease. In the LIVE-HCM cohort, ~ 5% of patients had a significant cardiac event. It is important for a family to understand that with or without competitive athletics, there is a risk of a catastrophic event.
3. Discuss current data. Current guidelines recommend using labs, CPET, echocardiography, cardiac MRI, ECG, and risk prediction scores. All this information should be disclosed to family, equipping them with the knowledge to make an educated decision on sport participation.
4. Provide exercise and athletic recommendations, aligning current data, clinical assessment of risk, and patient and family desires. If they are in collegiate or professional athletics, it is important to communicate these recommendations directly to the team physicians and training staff. Given the natural progression of HCM, most important is the need for continued follow-up with new data to reassess risk/benefit ratio of sport participation.

We also acknowledge that there are challenges to implementing extensive SDM discussions amid a busy clinic schedule. Assessing and normalizing stakeholder desires for sport participation can be emotional and time intensive. Communicating updated cardiac testing and risk stratification scores, as well as their limitations in predicting sudden death during exercise, is also time consuming. If the patient is a collegiate or professional athlete, additional stakeholders can include coaches, trainers, team physicians, agents, etc. Often, these people are not available during the time of the patient visit, so a physician distilling information relevant to these stakeholders may take additional time and energy. We recommend establishing a workflow that allows for all components of the SDM framework, which will be different for each center, but can include scheduled pre-visit testing where results are available at time of visit, longer initial visit lengths, and follow-up video visit specifically catered to coaches/trainers/agents.

In light of the changing attitudes toward exercise in children with HCM, several recent studies have evaluated the use of structured PA programs in this population. The Children’s Hospital of Philadelphia recently completed a 16-week pilot study of 8 pediatric patients with HCM (NYHA Class I and II) [28]. The intervention included three 30-minute aerobic sessions and two strength sessions per week. The patients were given a FitBit device and a target heart rate zone, guided by anaerobic threshold on a baseline exercise stress test. Exercise sessions were completed on the patients’ own time and recommendations were individualized based on available community resources (parks, gyms, or home exercise equipment). All subjects who completed the program demonstrated improved exercise performance and quality of life measures. Importantly there were no intervention-related arrhythmias or cardiac adverse events. This study, and similar adult studies [29], suggest that interventional exercise programs have the potential to be efficacious in children with HCM. Challenges remain, such as access to fitness centers, safe places to play outside, and food insecurity, but building healthy habits through a structured rehab program may be a good place to start developing healthy and safe PA habits in children with HCM.

## Future Directions and Unanswered Pediatric-Specific Challenges

Hypertrophic cardiomyopathy is a complex, evolving disease that incurs an increasing risk of sudden cardiac death over time. Historical guidelines were generally conservative with respect to PA in those with HCM. However, accumulating evidence suggests that vigorous exercise and competitive sports may not confer an increased risk of SCD in selected patients with HCM. Further, it appears that being sedentary has substantial negative effects on patients with HCM, including increased LV mass, acquired cardiovascular risk factors, and impaired emotional well-being. As such, guidelines have changed to allow for a more nuanced discussion of exercise and competitive sports in these patients. Studies over the past two years, including the LIVE-HCM cohort, continue to show safety of exercise for certain patients with HCM.

However, continued research is required to better understand patient-specific risk in children with HCM. Pediatric patients were the minority in LIVE-HCM (36% of the vigorous competitive athletes) and more studies are needed to understand this patient population. In this period of time when exercise restrictions are becoming less conservative, without fully understanding exercise-specific risk, secondary prevention with AEDs and EAPs are critical. Continued research is also needed to determine potential benefits of exercise. There is limited understanding about how exercise affects fibrosis progression, sarcomeric signaling pathways, and microvascular function. Furthermore, with the introduction of new therapeutic agents in pediatrics, such as myosin inhibitors [30], examination of the relationship of these medications to exercise capacity and PA related risk is warranted.

Lastly, and potentially most importantly, exercise programs and prescriptions should account for neighborhood and economic factors. Masood and colleagues have demonstrated that these factors are independently associated with lower exercise performance [20]. Attention should be paid to these disparities when creating supervised exercise programs and sports cardiology clinics. We hope the guidelines and new research cited in this paper promote safe PA for pediatric patients with HCM and drive continued research for this high-risk patient population.

## Key References

- Lampert R, Ackerman MJ, Marino BS, et al. Vigorous exercise in patients with hypertrophic cardiomyopathy. JAMA Cardiol. 2023;8(6):595. 10.1001/jamacardio.2023.1042.
  - LIVE-HCM cohort demonstrating vigorous exercise has no difference in death, ICD shock, resuscitated death, or arrhythmic syncope for HCM patients.
- Kim JH, Baggish AL, Levine BD, et al. Clinical considerations for competitive sports participation for athletes with cardiovascular abnormalities: A scientific statement from the american heart association and american college of cardiology. Circulation. 2025;151(11). 10.1161/CIR.0000000000001297.
  - The 2025 guidelines which promote consideration of competitive sports, after SDM and yearly evaluation.

## Data Availability

No datasets were generated or analysed during the current study.
